# Geolocation of unpublished archaeological sites in the Peruvian Amazon

**DOI:** 10.1038/s41597-021-01067-7

**Published:** 2021-10-29

**Authors:** Oliver T. Coomes, Santiago Rivas Panduro, Christian Abizaid, Yoshito Takasaki

**Affiliations:** 1 Department of Geography, McGill University, Montreal, QC Canada; 2 Universidad Nacional Mayor de San Marcos, Lima, Peru; 3 Department of Geography & Planning and School of the Environment, University of Toronto, Toronto, ON Canada; 4 Graduate School of Economics, University of Tokyo, Tokyo, Japan

**Keywords:** Archaeology, Databases

## Abstract

Published maps identifying archaeological sites in the Amazon basin show a paucity of sites in western Amazonia compared to the Brazilian Amazon. Whereas fewer than two dozen are identified for the Peruvian Amazon on basin-wide maps, a thorough review of unpublished archival material held by the Ministry of Culture of Peru and other sources revealed more than 400 known but unpublished sites in the Department of Loreto, challenging the notion that the region was sparsely occupied in prehistory. Our database provides the geolocation of each site and corresponding references for use by scientists seeking to better understand regional Pre-Columbian human occupation and settlement, cultural change, resource use and their landscape legacies. These data are foundational not only to the development of a richer understanding of prehistory and historical ecology of the Amazon basin but importantly for informing current land use, forest conservation and development policies as well as initiatives to support indigenous land and cultural rights in Amazonia.

## Abstract (summary)

**Table:** 

Measurement(s)	archaeological site • geographic location
Technology Type(s)	digital curation
Sample Characteristic - Location	Amazon Basin

Machine-accessible metadata file describing the reported data: 10.6084/m9.figshare.16750639

## Background & Summary

An important ongoing debate in Amazonian studies is the extent to which indigenous peoples occupied and transformed their environment in prehistory. Evidence from the Brazilian Amazon – from Pre-Columbian geoglyphs, settlement sites, raised fields, and causeways – points to large complex polities in nucleated and interconnected settlements along the rivers and interfluves^1–5^. The discovery of extensive anthropic soils (*terra preta*)^6,7^, the enrichment of forests with useful and semi-domesticated species^8–12^, and the presence of landscape-scale fisheries infrastructure^13^ together suggest enduring landscape transformations by purposeful human action over millennia^14–19^. Still, some scientists remain sceptical of the extensive human modification thesis, pointing to contrary evidence drawn often from beyond Brazil, in the outer Amazon basin [e.g.^20–22^].

**Fig. 1:**
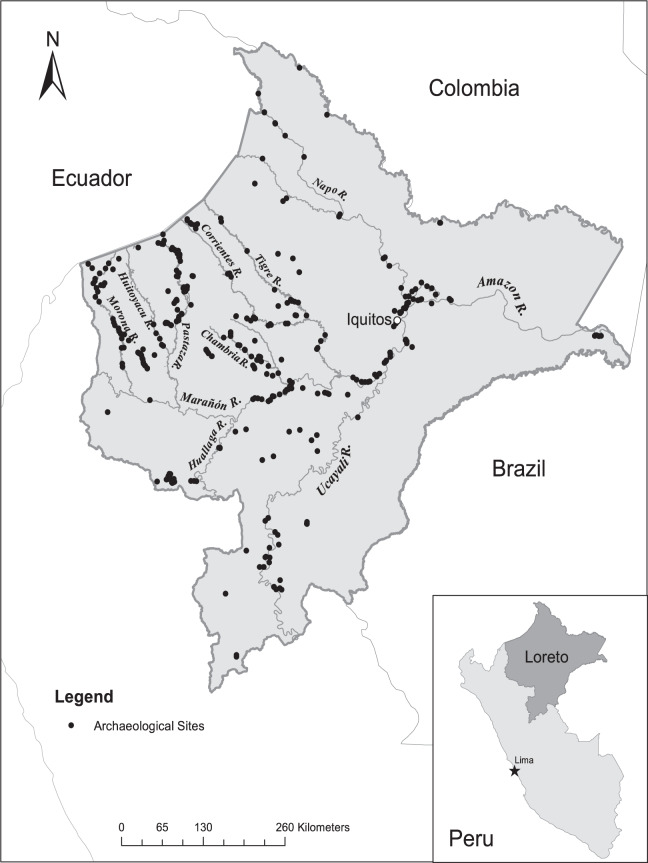
Archaeological sites in the Department of Loreto, Peruvian Amazon.

Western Amazonia remains far less studied by using controlled archaeological investigative methods than Brazil^23^. Published maps identify fewer than two dozen sites in the Peruvian Amazon [see^20^: Fig. 1^23^; Fig. 2.2.2^24^: p. 175;^25^: Map 1;^26^: Fig. 1]. Other evidence of human occupation also remains scant, particularly *terra preta* soils which have been rarely reported in Peru^27–29^– the same can be said for geoglyphs, petrographs, monuments and earthworks. To some scientists, the paucity of sites and supporting phytolith evidence in the Peruvian Amazon suggests that human occupation and impacts on the forests and land in pre-Columbian times were much more limited than in Brazil [see^20^].

In addition to the typical challenges to *in situ* conservation of cultural material in humid forest tropical soils, the lowlands of the Peruvian Amazon have some of the most active meandering rivers in the world^30,31^, making it difficult to locate and preserve archeological sites. In an influential article, Donald Lathrap identified two primary threats to prehistoric sites along the major rivers – floodplain aggradation which drowns sites; and, rapid river meandering destroys floodplain sites and erodes river bluffs^32^. Lathrap’s view of the salience of site instability due to river dynamics has been echoed by his students to explain the paucity of prehistoric settlement^33^ and *terra preta* soils^27^, and may have dampened international interest in the pursuit of archaeological sites in the Peruvian Amazon. Nevertheless, many propitious locations remain, on river terraces or bluffs^34^. Two recent reviews of archaeological evidence along the Napo River^35^ and Lower Ucayali River^36^ identify many more sites than previously reported in the literature.

**Fig. 2:**
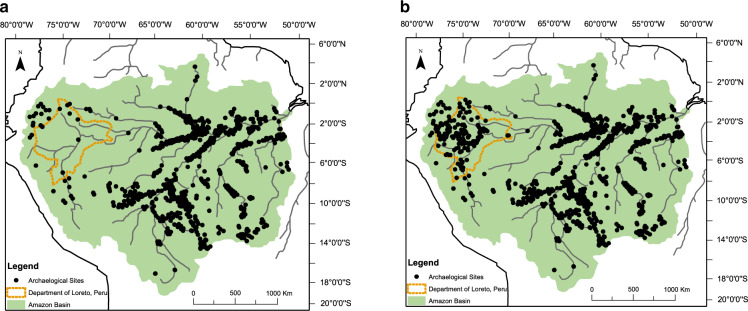
Archaeological sites in the Amazon basin according to^5^. (**a**) Original map (adapted from^5^). (**b**) Map including unpublished sites in the Department of Loreto.

**Fig. 3:**
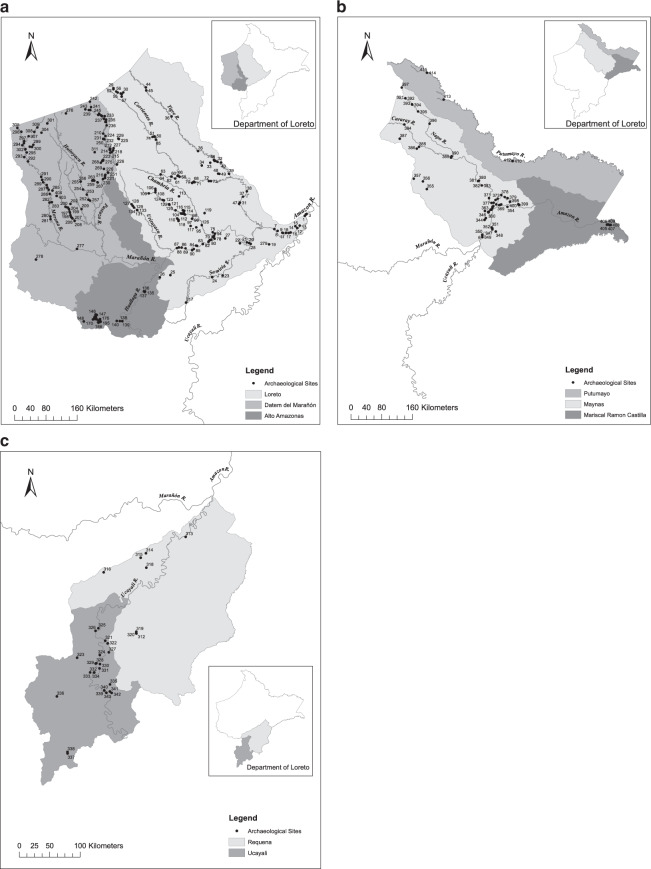
Archaeological sites in the Department of Loreto, Peruvian Amazon by province. (**a**) Loreto, Datem del Marañón and Alto Amazonas; (**b**) Putumayo, Maynas and Mariscal Ramon Castilla; and, (**c**) Requena and Ucayali.

Our database comprises a listing of 415 archaeological sites documented to date in the Department of Loreto in the Peruvian Amazon. Loreto extends over a vast area–of 368,851 km^2^–and is the largest department by area in the Peruvian Amazon. For each site we provide the longitude and latitude of the site location and the corresponding key reference(s). We have summarized and mapped the distribution of sites in Loreto (Fig. 1; Table 1) and compare them to a recently published basin-wide map to show a much higher density of archaeological sites in Peru than is recognized among scientists working in the Amazon basin (see Fig. 2a,b). In addition, we have mapped the sites by province within the Department of Loreto (see Fig. 3a–c). We consider the number of sites reported to be the minimum number known; other known but unreported sites are likely to exist, and still many others remain to be documented.

**Table 1:** Distribution of archaeological sites (n = 415) by province and district in the Department of Loreto, Peruvian Amazon.

**Province**		
***Alto Amazonas***		
**District**	**Number**	**Percent**
Balsapuerto	56	90
Lagunas	3	5
Yurimaguas	3	5
Total	62	100
Percent of Total in Loreto	15%	
***Datem del Marañón***		
**District**	**Number**	**Percent**
Andoas	68	59
Barranca	2	2
Manseriche	1	1
Morona	30	26
Pastaza	14	12
Total	115	100
Percent of Total in Loreto	28%	
***Loreto***		
**District**	**Number**	**Percent**
Nauta	19	14
Parinari	9	7
Tigre	21	16
Trompeteros	25	19
Urarinas	60	45
Total	134	100
Percent of Total in Loreto	33%	
***Requena***		
**District**	**Number**	**Percent**
Emilio San Martín	3	33
Puinahua	5	56
Requena	1	11
Total	9	100
Percent of Total in Loreto	2%	
**Province**		
***Ucayali***		
**District**	**Number**	**Percent**
Alfredo Vargas Guerra	4	17
Contamana	6	26
Emilio San Martín	2	9
Inahuaya	4	17
Pampa Hermosa	1	4
Puinahua	1	4
Sarayacu	5	22
Total	23	100
Percent of Total in Loreto	6%	
***Maynas***		
**District**	**Number**	**Percent**
Alto Nanay	3	5
Fernando Lores	8	14
Indiana	5	8
Las Amazonas	5	8
Mazán	10	17
Napo	11	19
Punchana	7	12
San Juan Bautista	3	5
Torres Causana	7	12
Total	59	100
Percent of Total in Loreto	14%	
***Mariscal Ramón Castilla***		
**District**	**Number**	**Percent**
Ramón Castilla	5	71
San Pablo	2	29
Total	7	100
Percent of Total in Loreto	2%	
***Putumayo***		
**District**	**Number**	**Percent**
Putumayo	3	50
Teniente Manuel Clavero	3	50
Total	6	100
Percent of Total in Loreto	1%

Our documentation of hundreds of archaeological sites in the Peruvian Amazon challenges the notion of sparse early human occupation in the outer Amazon basin and provides the basis for a richer understanding of the past as well as for informing current issues regarding land use, conservation and development as well as indigenous claims and rights to the land and their cultural heritage. These data can serve to guide archaeologists, ethnobotanists, cultural anthropologists, geographers, heritage specialists, indigenous scholars, palynologists, soil scientists and forest ecologists in research on human settlement, cultural change, resource use, plant domestication and landscape transformation in western Amazonia. Policy makers will also benefit from knowledge of the existence and location of these sites for land use and development planning (including site preservation) as will indigenous peoples and the groups that support them through initiatives to buttress indigenous land and cultural rights in Amazonia.

## Methods

The archaeological site database was constructed based on a thorough review of published reports in the international and national literatures as well as unpublished materials held by the *Ministerio de Cultura* (formerly *Instituto Nacional de Cultura*) in Iquitos and Lima and other institutions. These sites were progressively catalogued by Santiago Rivas Panduro (SRP, now deceased), an archaeologist who worked in Iquitos for the *Ministerio de Cultura* during the early 2000s and later as a consulting archaeologist. Very few of the sites have yet been subjected to controlled investigation and most references are found in unpublished site visit and reconnaissance reports, environmental impact statements, conference presentations, and oral testimonies. Many sites were encountered by accident, often during road building and petroleum exploration activities including the running of seismic lines, road construction, and well drilling. SRP was responsible for documenting many of the sites reported through field reconnaissance and this database represents the culmination of his career’s work.

## Data Records

The database is stored at figshare as an Excel worksheet^37^. Each record corresponds to one archaeological site and comprises 12 fields: (1) Map site number; (2) Site name; (3) Site code; (4) Department; (5) Province; (6) District; (7) Longitude; (8) Latitude; (9) Elevation (Google Earth); (10) Elevation (Recorded); (11) Site Location notes (Satellite imagery 2020); and (12) Reference(s). The “Map site number” corresponds to the number we assigned to identify the site on our provincial maps (Fig. 3a–c). The “Site name” is recorded as per the citation in the corresponding Reference(s) where the site is mentioned and is often the name of the nearest human settlement, landmark, well site or facility (e.g., military base). The “Site code” refers to the identifying name given to the site. For sites registered with the *Ministerio de Cultura*, the code refers to the department (e.g., LOR, for Loreto), river (e.g., COR, for Corrientes River), and a site number. Unregistered sites retain the code given by the reporting archaeologist (e.g., UCA-29, Myers 1967). “Department”, “Province”, and “District” record the administrative units in which the site is located (e.g., Loreto; Maynas; Fernando Lores). “Longitude” and “Latitude” are recorded in decimal degrees and are approximate only, except where indicated as being measured on-site; “On-site Measurement” indicates whether longitude and latitude were measured with a GPS on site (yes/no). “Elevation (Google Earth)” is recorded in metres above sea level (asl) according to Google Earth and as such are approximate. “Elevation (recorded)” captures the elevation as measured on-site with a GPS. “Site Location notes (Satellite imagery 2020)” refers to any observations made regarding site location based on verification using Google Earth imagery in November 2020. “Reference(s)” refers to the documents that correspond to the site, typically a project report, impact statement, or publication. We present citations in the original language of the text.

## Technical Validation

The data were compiled over a period of nearly 20 years by a professional archaeologist (SRP) who was considered by his international peers to be the leading archaeologist in the Department of Loreto of Peru. Each site reported was confirmed with a corresponding citation to a published or unpublished work(s). Of the 415 sites, 36% are registered with the *Ministerio de Cultura* and an additional 13% have a formal site code. In November 2020, the accuracy of each site location was checked using Google Earth imagery. Of the 415 sites, 22 sites (5%) were found now to lie in the active channel of the nearby river, reflecting changes in the river course since the site was originally reported; 29 sites are now located on the river’s edge. A total of 163 sites were documented as part of impact assessments and field monitoring of oil exploration and road building activities, at least some of which may have been destroyed in the development process.

## Usage Notes

Our database provides high quality geolocational data for scientists, researchers, planners, policy makers, government agencies, and indigenous peoples to locate over 400 unpublished archaeological sites in the Peruvian Amazon. Locational data contained in this database are intended for re-use in at least three realms.

1. *Archaeology*. The database will advance the field of Amazonian archaeology by saving archaeologists’ considerable time and effort in locating sites and locales of potential interest and significance. The location data can be used to guide future archaeological investigations using remote sensing analyses (e.g., Lidar) and controlled field excavations to document extant and undocumented archaeological sites; in the development of a more complete cultural chronology and understanding of the historical ecology of Amazonia; to protect and preserve archaeological sites under threat of destruction; to inspire, guide and train a new generation of Peruvian archaeologists and stimulate archaeological research in western Amazonia; and, to valorise the cultural heritage of indigenous peoples in this less studied but extensive region of the Amazon basin. The database could serve as a template for the development of similar compilations for other Amazonian countries as well as a basin-wide database of documented archaeological sites.
2. *Development activity impacts and land use planning*. The site location data can be used to assess the potential impacts of development activities in the Peruvian Amazon, a resource frontier region with significant exploration and production of oil, gas, gold and timber and attendant road building. Knowledge of where archaeological sites are located will inform decision makers as to the need for impact assessments and protective/mitigative measures. Regional and local governments as well as local communities can use these data in the formulation of land use planning policy, conservation and development plans in the Peruvian Amazon. Location data will also be central to the Ministry of Culture’s efforts to protect and preserve archaeological sites from immanent development and environmental threats.
3. *Indigenous land claims and cultural rights*. Indigenous peoples and advocacy groups can use the data to legitimize and buttress land claims and cultural rights in Amazonia. The paucity of documented archaeological sites is often used as evidence of a lack of indigenous occupation and thus (pre)historical rights to land and other resources. Indigenous federations, NGOs, scientists and scholars (including indigenous scholars) will benefit significantly by knowledge of the location of the many archaeological sites referenced in the database. These data may also be used to empower individual communities in their struggle to protect their lands from invasion.

This database is limited in three respects that are important to acknowledge but which do not diminish the quality or re-use value of the data.

1. *Potential site destruction*. Some sites identified in the database are prone to destruction by fluvio-geomorphological change and/or development activities and thus may no longer exist. Most archaeological sites located along or near rivers which vary in their exposure to riverbank erosion and aggradation. Of the 415 sites verified in November of 2020, 22 sites (5%) now lie in the active channel of a river, reflecting changes in the river course since the site was originally reported; 29 sites are currently located on the river’s edge. Site instability means that reported sites may not persist in the future. The same can be said of sites documented as part of impact assessments and field monitoring conducted for oil exploration and road building activities (n = 163). Although one of the purposes of assessments and monitoring is to identify archaeological sites for preservation/conservation, it is possible that a small number of sites may have been further disturbed by subsequent development activities or destroyed. Publication of the database is urgently needed to protect and preserve known sites.
2. *Un-documented sites*. The list of archaeological sites reported in the database for the Peruvian Amazon is partial only, as we anticipate that more sites remain to be documented through further field reconnaissance and excavation. Still, the current listing of georeferenced unpublished sites reported is the most comprehensive to date; is much more extensive than records available in the published literature from the Peruvian Amazon; demonstrates clearly how the region is far from impoverished in terms of archaeological sites and cultural prehistorical heritage; and, suggests the promise for new sites remain to be encountered and documented.
3. *Limited site descriptive information*. Limited information is provided in the database of site characteristics, archaeological tradition or phase, or material culture which would be a valuable supplementary addition through future work. By providing the site location, name and registry information, database users can access more detailed site information from the corresponding citations reported for each site and pursue further site exploration and excavation as might be of interest. Of the 415 sites, 36% are registered with the Ministry of Culture and additional 13% have a formal site code. Database users can refer to the offices of the Ministry of Culture in Lima and Iquitos for access to corresponding documentation for sites registered with the Ministry.

## Data Availability

No custom code was used to generate or process the data.
